# Effects of Ultraviolet (254nm) Irradiation on Egg Hatching and Adult Emergence of the Flour Beetles, *Tribolium castaneum*, *T. confusum* and the Almond Moth, *Cadra cautella*

**DOI:** 10.1673/031.007.3601

**Published:** 2007-05-29

**Authors:** S. I. Faruki, D. R. Das, A. R. Khan, M. Khatun

**Affiliations:** Department of Zoology, Rajshahi University Rajshahi, 6205, Bangladesh

**Keywords:** physical control, red flour beetle, confused flour beetle, almond moth, tropical warehouse moth

## Abstract

The eggs of the stored grain pests, *Tribolium castaneum* (Herbst), *T. confusum* (Duval) (Coleoptera: Tenebrionidae) and *Cadra cautella* (Walker) (Lepidoptera; Pyralidae) belonging to three age groups, 1, 2, and 3 days-old, were exposed to ultraviolet (UV) radiation with 254nm wavelength (UV-C) for different durations to determine irradiation effects on egg-hatching and adult emergence. An increase in time of exposure to UV-rays caused a gradual decrease in the percentage of hatching of eggs in all age groups of eggs. No hatching occurred after 24 minutes of exposure in 2 and 3 day-old eggs of *T*. *confusum. C. cautella* eggs were less sensitive to UV-rays than were *T. castaneum* and *T. confusum* eggs. All the exposure periods significantly reduced the eclosion of adults in all the experimental insects. No adults emerged when 3 day-old eggs of *T. castaneum* were irradiated for 16 or 24 minutes, or from 2 and 3 day-old eggs *T. confusum* irradiated for 16 or 24 minutes.

## Introduction

The presence of insects in stored products results in both contamination and substantial economic damage due to the loss of the products and a decrease of nutritional value (Wilbur and Mills 1985; Burkholder and Faustini 1991). Fumigants and other chemical insecticides are widely used to protect stored commodities from insect infestations and contamination but their use leads to problem of undesirable residues (Anon. 1974, 1976; Mensah et al. 1979) and development of resistance in certain insect species (Champ and Dyte 1976, 1977; Zettler 1982; Saleem and Shakoori 1989, 1990). Moreover, the injudicious use of synthetic pesticides and its concomitant impact on environment has necessitated exploration for alternative non-toxic pest control methods. Irradiation has become an established technique for controlling stored grain insects because of residue free advantages over chemical fumigation (Tuncbilek 1995). Pszczola (1997) demonstrated the acceptability of irradiation technology as an alternative treatment for food protection because irradiation can extend the shelf life of various fruits and vegetables, and maintain the quality of the product over a longer period of time. Irradiated foods may be more acceptable to those sensitive to chemical treatments (Pszczola 1997). Hasan and Khan (1998) mentioned that irradiation does not significantly change the quality of the food material or stored seeds. Generally, ionizing radiation such as gamma rays and X-rays are used for the disinfestations of bulk grains under storage conditions. Treatment with UV-radiation has obviously less penetrating effect than ionizing radiations and therefore has limited use for bulk grains. However, the results have theoretical value and UV-radiation can be used for the treatment of small amounts of grains intended for household use.

The Ultraviolet (UV) portion of the spectrum is widely used as germicide and as an attractant for insects (Bruce 1975), in embryological-physiological studies (Bodenstein 1953) and for the surface disinfection of insect eggs (Guerra et al. 1968). A number of investigators have considered the possibility of using UV-rays to control, or at least to suppress development of various species of stored products insects (Calderon and Navarro 1971; Calderon et al. 1985; Faruki and Khan 1993; Sharma and Dwivedi 1997; Faruki 2005; Faruki et al. 2005).

Typically, the embryonic stage of an animal is a period of higher radio sensitivity and insects are no exception (Tilton and Brower 1983). The rust-red flour beetle, *Tribolium castaneum* (Herbst), the confused flour beetle, *T. confusum* duVal, and the almond moth, *Cadra cautella* (Walker), also known as the tropical warehouse moth, are major storage pests. The present investigation was, therefore, aimed at determining the effects of UV-rays (UV-C) on hatching and adult emergence in *T. castaneum*, *T*. *confusum* and C. *cautella* eggs of various ages.

## Materials and Methods

### Sources and rearing of test insects

*T. castaneum* was originally received from the Pest Infestation Control Laboratory, Slough, England, and *T. confusum* was obtained from the Food Entomology Laboratory, National Food Research Institute, Tsukuba, Japan. They were cultured for a few years in the Insect Research Laboratory, Department of Zoology, University of Rajshahi, on a rearing medium containing whole-wheat flour and Brewer's yeast (19:1). *C*. *cautella* was collected from a Government Warehouse at Rajshahi as larvae and reared on groundnut kernels (*Arachis hypogaea* L.).

### Egg production, collection and sorting of eggs

The eggs of *Tribolium* spp. were collected by placing large numbers of beetles on the rearing medium. On the following day the culture medium was sieved to separate the adults and eggs that were 1 day-old. The eggs were kept in Petri dishes to obtain the eggs of 2 and 3 day-old eggs.

For collecting the eggs of *C*. *cautella*, a number of healthy pupae were sexed and kept in beakers until adult eclosion. Freshly emerged moths were allowed to mate and placed in beakers for oviposition. A piece of hard white paper was inserted inside the beaker for resting of the adults and the top of the beaker was covered with a netted cloth. The beakers with moths were inverted over Petri dishes for an easy collection of the eggs. On the following day 1 day-old eggs were collected. Some of these eggs were kept in Petri dishes to obtain 2 and 3 day-old eggs.

The incubation period of the eggs of *T. castaneum* and *T. confusum* varies from 6–7 days and of *C*. *cautella* from 5–6 days at 30° C.

**Table 1.**
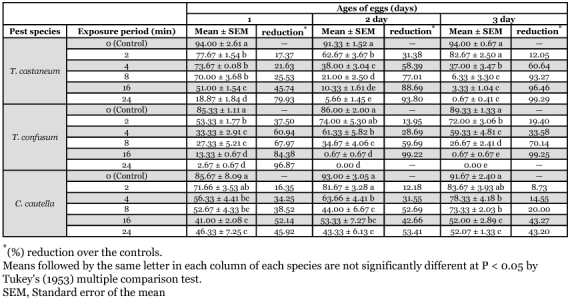
Hatching (%) of UV-irradiated eggs of three stored products insects

### UV-irradiation technique of eggs

The aim of these experiments was to determine age-specific resistance of the developing eggs to UV-irradiation. The experiments were conducted at a mean room temperature of 30 ± 2° C under an illumination of two 40W fluorescent bulbs with a photoperiod of 10:14 L:D without any humidity control.

A 15W UV germicidal lamp (GE15T8)(F.G. Bode & Co. Gmbh, www.optiker-bode.de) measuring 20 cm × 4 cm, emitting at a wavelength of 254nm (UV-C) was only the source of irradiation. During irradiation the room was illuminated with two 40W fluorescent bulbs. For irradiation of *T. castaneum* and *T. confusum*, eggs in a Petri dish (9cm) were placed on a surface (20.5 cm^2^) 12 cm from the lamp. Eggs were irradiated for 2, 4, 8, 16 or 24 minutes. Exposure period was determined using a stopwatch. At the end of the exposure period the UV-lamp was turned off and the Petri dishes were removed immediately. After exposure, the irradiated and non-irradiated control eggs of different age groups were kept separately at 30 ± 2° C until hatching. Neonate larvae from both irradiated and non-irradiated eggs were transferred to plastic containers with rearing medium to observe adult eclosion. The experiment was replicated 3 times with 50 eggs for each exposure period and an equal number of non-irradiated eggs were raised as controls for each age group.

*C*. *cautella* eggs of desired ages were irradiated similar to the procedure of *Tribolium* eggs. Eggs of different ages and exposure periods, either irradiated and/or non-irradiated, were kept in separate pieces of black adhesive tape to observe hatching. The black tape was used to prevent the hatched larvae from crawling away. To observe the emergence of adults, eggs were kept in separate Petri dishes with groundnut kernels until adult eclosion. All the experiments including controls for each age group were replicated 3 times with 100 eggs each.

### Data and statistical analyses

Data were analyzed by Factorial analysis of variance (ANOVA) using Minitab Inc. and a comparison test was done between means of control and exposure periods by Tukey's test of multiple comparisons (1953). The percent reduction in hatching of eggs in comparison to control was calculated using the formula: Percent reduction = (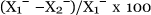, where, 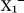 = mean hatching of control eggs and 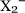 = mean hatching of irradiated eggs. The adult recovery (%) was also analyzed by computing standardized normal deviate values (d, the difference between the mean and the value of interest divided by the standard deviation) for determining the differences in parameters having binomial distribution.

## Results

### Effect on hatching

It was found that UV-irradiation reduced hatching of eggs of all age groups; the effect gradually increased with increasing exposure periods (Table 1). Factorial ANOVA on hatching of eggs showed that UV-rays had significant effects on all species and their interactions (Table 3). All exposure periods of UV-radiation reduced the hatching of eggs in comparison to controls. It was observed that older eggs (2 and 3 day-old) of *T. castaneum* and *T. confusum* were more sensitive to UV-rays than younger eggs (1 day-old). Table 1 shows that in *T. castaneum* hatching of 1, 2 and 3 day-old eggs were reduced to 80, 94, and 99% respectively, after 24 min of exposure to UV-radiation. Hatching was inhibited up to 97, 100 and 100% respectively by 24 min exposure of 1, 2 and 3 day-old eggs of *T. confusum* to UV-irradiation. On the other hand, in *C*. *cautella* the younger eggs (1 and 2 day-old) were more sensitive to UV-rays than older eggs (3 day-old) (Table 1). The 1 day-old eggs of *T. castaneum* were less sensitive to irradiation than 3 day-old eggs, but the 1 day-old eggs of *T. confusum* and *C. cautella* were more sensitive. At all ages and exposure periods, *C. cautella* eggs were less sensitive to UV-rays than were *T. castaneum* and *T. confusum* eggs (Table 1).

**Table 2.**
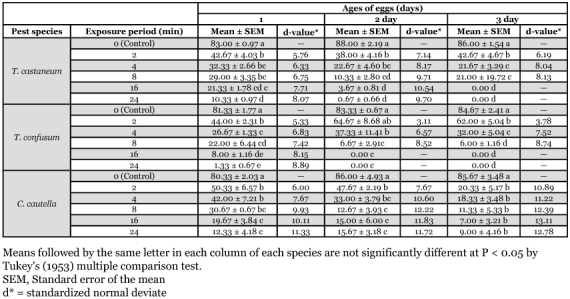
Adult emergence (%) from UV-irradiated eggs of three stored products insects

### Effect on adult emergence

Adult eclosion from UV-irradiated eggs of *T. castaneum*, *T. confusum and C. cautella* gradually decreased as the duration of exposure to radiation increased (Table 2). Very few adults emerged from 2 day-old eggs of *T. castaneum* and no adults developed from 3 day-old eggs of *T*. *castaneum* and 2 and 3 day-old eggs of *T. confusum* after 16 and 24 minutes of exposure to UV. In *C*. *cautella,* 3 day-old eggs were most affected by exposure to UV for 24 minutes. Statistical analyses showed that exposure periods to UV-irradiation significantly reduced adult emergence, but there were no significant differences between the species in adult emergence following irradiation (Table 3).


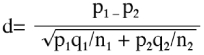


## Discussion

A significant reduction in egg hatching as well as in adult emergence of three experimental storage insects was observed when eggs of different ages were exposed to UV irradiation. The results also showed that the younger eggs of *Tribolium* spp. and *C*. *cautella* were more sensitive to UV-rays than older ones that contrast with the findings of Calderon and Navarro (1971) who observed that older eggs of *Ephestia* (=*Cadra*) *cautella* were highly sensitive to UV-rays than younger eggs. Guerra et al. 1968 reported that when eggs of *Heliothis virescens* and *H. zea* were exposed to UV-rays of a short wavelength (2537A°) the percentage of egg-hatch was gradually decreased with increasing time of exposure, and no hatch occurred after an exposure of 20 minutes. Faruki et al. 2005 recorded that the fecundity and fertility of *Alphitobius diaperinus* eggs resulting from UV-irradiated 2^nd^ and 3^rd^ instar larvae were reduced significantly. Hasan et al. 1998 observed reduced fertility in eggs of the Uzi fly, *Exorista sorbillans* developing from UV-irradiated pupae. The present results support the finding of Beard (1972) who noted that eggs of the Indianmeal moth, *Plodia interpunctella*, the wax-moth, *Galleria mellonella*, the large milkweed bug, *Oncopeltus fasciatus*, and worker termites were remarkably sensitive to UV-irradiation.

**Table 3.**
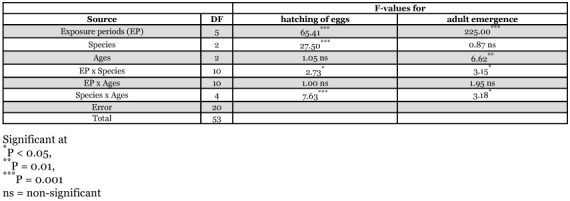
Factorial ANOVA on hatching of eggs and adult emergence from UV-irradiated eggs of three stored products insects

Yang and Sacher (1969) irradiated *T. castaneum* eggs of various ages with X-rays to determine the effect of doses and age on hatching. They recorded a delay in development that was proportional to the irradiation doses. The relation of the delay in development with respect to the dose was linear at all ages. Quraishi and Matin (1963) suggested that the sensitivity of eggs of the pulse beetle, *Callosobruchus chinensis*, to radioisotopes differed at different stages of their development. The work of Seidel et al. 1940 may give a possible explanation of higher sensitivity of the older eggs to UV-rays than the younger eggs. They found that during early embryonic organization injury to the peripheral parts of the eggs by UV-exposure did not impede the viability of the activation centre. As development proceeds the embryonic regions became more specialized, and different organ fields can no longer replace each other. Thus, damaging of the surface tissue of the eggs can be fatal at the advanced stages of development by non-penetrating radiations like UV-rays.

Similar reduction in adult eclosion was reported by Hasan et al. 1998 working with UV-irradiated pupae of *E. sorbillans*. The present findings are also similar to the findings of Beard (1972), who reported that adult emergence was progressively decreased by higher doses when late stage larvae of *P. interpunctella* were irradiated with UV-rays. Adult emergence was significantly decreased when larvae of *T. castaneum* (Faruki 2005) and *A. diaperinus* (Faruki et al. 2005) were exposed to UV irradiation. When adult *C. chinensis* (Sharma and Dwivedi 1997) and pupae of *A. diaperinus* (Parween et al. 2004) were exposed to UV-irradiation, the production of adult progeny was gradually reduced.

The significantly reduced hatching and adult emergence caused by UV-irradiation in the experimental pests is promising from pest management point of view. It may be concluded that irradiation is a very safe and clean method for food preservation and pest control. However, much more comprehensive research is needed.

## References

[bibr01] Anonymous. 1974 Evaluations of some pesticide residues in food. *FAO, AGP: 1974/M/11; WHO pesticide Residue Ser. No*. 4 545

[bibr02] Anonymous. 1976 Pesticide residues in food. Report of the 1975 Joint Meeting of the FAO Working Party of Experts on Pesticide Residues and WHO Expert Committee on Pesticide Residues. *FAO Plant Prod. Protect. Ser. No. 1; WHO Tech. Rep. Ser. No. 592.* 45 821259

[bibr03] Beard RL 1972 Lethal action of UV-irradiation on insects. *Journal of Economic Entomology* 65 650 654 5028267 10.1093/jee/65.3.650

[bibr04] Bodenstein D 1953 Embryonic development. Roder KD *Insect Physiology.* 780 822 John Wiley

[bibr05] Bruce WA 1975 Effect of UV-radiation on egg hatch *of Plodia interpunctella* (Lepidoptera: Pyralidae). *Journal of Stored Products Research* 11 243 244

[bibr06] Burkholder WE Faustini DL 1991 Biological methods of survey and control. Gorham JR *Ecology and Management of Food Industry Pests.* 361 372 AOAC press

[bibr07] Calderon M Navarro S 1971 Effects of ultra-violet irradiation on the eggs of *Ephestia cautella* (Wlk) (Lepidoptera: Phycitidae). *Journal of Stored Products Research* 7 309 311

[bibr08] Calderon M Bruce WA Leesch LG 1985 Effect of UV-radiation on eggs of *Tribolium castaneum*. *Phytoparasitica* 13 179 244

[bibr09] Champ BR Dyte CE 1976 Report of the FAO global survey of pesticides susceptibility of stored grain pests. *FAO Plant Production and Protection Series No. 5.* 297 Food and Agriculture Organization of the United Nations

[bibr10] Champ BR Dyte CE 1977 FAO global survey of pesticide susceptibility of stored grain pests. *FAO Plant Protection Bulletin* 25 49 67

[bibr11] Faruki SI 2005 Effects of UV-radiation on the growth and development of malathion-susceptible and multi-resistant strains of *Tribolium castaneum* (Herbst) (Coleoptera: Tenebrionidae). *Bangladesh Journal of Entomology* 25 55 63

[bibr12] Faruki SI Khan AR 1993 Potency of UV-radiation on *Carda cautella* (Walker) (Lepidoptera : Phycitidae) larvae treated with *Bacillus thuringiensis* var. *kurstaki*. *University Journal of Zoology*, *Rajshahi University* 12 73 79

[bibr13] Faruki SI Das DR Khatun S 2005 Effects of UV-radiation on the larvae of the lesser mealworm, *Alphitobius diaperinus* (Panzer)(Coleoptera: Tenebrionidae) and their progeny. *Pakistan Journal of Biological Sciences* 5 444 448

[bibr14] Guerra AA Ouye MT Bullock HR 1968 Effect of ultraviolet irradiation on egg hatch, subsequent larval development, and adult longevity of the tobacco budworm and the bollworm. *Journal of Economic Entomology* 61 541 542

[bibr15] Hasan M Khan AR 1998 Control of stored-product pests by irradiation. *Integrated Pest Management Review* 3 15 29

[bibr16] Hasan M Jahan MS Khan AR 1998 Effect of UV-radiation on the Uzi-fly, *Exorista sorbillans* Widemann, an endoparasitoid of the silkworm, *Bombyx mori* L. *Insect Science & its Application* 18 87 91

[bibr17] Mensah GWK Watters FL Webster GRB 1979 Insecticide residues in milled fractions of dry or tough wheat treated with malathion, bromophos, iodofenphos, and pirimiphos-methyl. *Journal of Economic Entomology* 72 728 731 544641 10.1093/jee/72.5.728

[bibr18] Parween S Faruki SI Akther R 2004 Growth and development of *Alphitobius diaperinus* (Panzer) (Coleoptera: Tenebrionidae) developing from pupae irradiated with ultra-violet rays. *University Journal of Zoology*, *Rajshahi University* 23 23 26

[bibr19] Pszczola DE 1997 20 ways to market the concept of food irradiation. *Food Technology* 51 46 48

[bibr20] Quraishi MS Matin M 1963 Radiosensitivity of various stages of *Callosobruchus chinensis* L. *Radiation and radioisotopes applied to insects of agricultural importance.* 479 484 IAEA

[bibr21] Saleem MA Shakoori AR 1989 Toxicity of malathion, permethrin, and cypermethrin against resistant and susceptible strains of *Tribolium castaneum* (Herbst.). *Pakistan Journal of Zoology* 20 347 360

[bibr22] Saleem MA Shakoori AR 1990 The toxicity of eight insecticides to sixth instar larvae and adult beetles of *T*. *castaneum* (Herbst.). *Pakistan Journal of Zoology* 22 207 216

[bibr23] Seidel F Bock E Krause G 1940 Die organisation des insekteneis. *Naturwissenschaften* 28 433 446

[bibr24] Sharma MK Dwivedi SC 1997 Investigation on the effects of ultraviolet and infrared light on the life cycle of *Callosobruchus chinensis* Linn. *Journal of Advanced Zoology* 18 27 31

[bibr25] Tilton EW Brower JH 1983 Radiation effects on arthropods. Josephson ES Peterson MS *Preservation of food by ionizing radiation* 2 269 316 CRC Press Inc

[bibr26] Tukey JW 1953 Multiple Comparisons. Zar JH *Biostatistical Analysis*4th edition Chapter 11. Prentice Hall International, Inc

[bibr27] Tuncbilek AS 1995 Effect of 60Co gamma radiation on the rice weevil, *Sitophilus oryzae* (L.). *Anzeiger fur Schadlingskunde Pflanzenschutz Umweltschutz* 68 37 38

[bibr28] Wilbur DA Mills RB 1985 Stored Grain Insects. Pfadt RE *Fundamentals of Applied Entomology*4th edition 552 576 Macmillan

[bibr29] Yang TCH Sacher GA 1969 Effects of X-irradiation on some physiological properties of a developing *Tribolium.* *Argonne National Laboratory Annual Report.* 49 50

[bibr30] Zettler JL 1982 Insecticide resistance in selected stored product insects infesting peanuts in the southern United States. *Journal of Economic Entomology* 75 359 362

